# Artificial Intelligence for Cardiac Ischemia Detection: A Review of Biosensor, Biomarker, and Imaging Applications

**DOI:** 10.3390/s26206503

**Published:** 2026-10-14

**Authors:** James W. H. Choi, Omar Seyam, Dhruva Biswas, Sanjay Sivalokanathan, Hua-Hsin Tai, Darren Kong, Serdar Farhan

**Affiliations:** 1 Northwell, Cardiovascular Institute, New Hyde Park, NY 11030, USAdkong@northwell.edu (D.K.); sfarhan1@northwell.edu (S.F.); 2 School of Cardiovascular and Metabolic Medicine and Sciences, The James Black Centre, King’s College London, London SE5 9NU, UK; dhruva.biswas@kcl.ac.uk; 3 Ataraxis AI, New York, NY 10002, USA; 4 Department of Cardiology, Icahn School of Medicine at Mount Sinai, Mount Sinai Morningside, New York, NY 10025, USA; sanjay.sivalokanathan@gmail.com; 5 Department of Clinical Informatics, Icahn School of Medicine at Mount Sinai, Mount Sinai Hospital, New York, NY 10029, USA; hua-hsin.tai@mountsinai.org

**Keywords:** artificial intelligence, deep learning, cardiac ischemia, electrocardiogram, biosensor, cardiac imaging, foundation models, myocardial infarction, risk stratification

## Abstract

Ischemic heart disease is the leading cause of global mortality, yet accurate and timely detection remains constrained by incomplete signal capture, noise, operator dependence, and imperfect reference standards. Artificial intelligence (AI) may enhance cardiovascular sensing by extracting latent diagnostic information from routinely acquired signals. This narrative review examines AI-enabled approaches to ischemia detection across three sensing modalities: the electrocardiogram as a bioelectric sensor, cardiac troponin as a biochemical marker, and cardiac imaging technologies (echocardiography, computed tomography, magnetic resonance, and nuclear imaging) as structural and functional sensors. For each modality, we evaluate the sensing pipeline, the reference standards, the endpoint actually measured (from acute coronary occlusion to functional ischemia), and reported performance metrics, separating diagnostic accuracy from effects on clinical workflow, patient outcomes, and cost. We also discuss emerging directions, including foundation models and multimodal fusion, together with translational challenges such as limited prospective validation, regulatory hurdles, and uncertain cost-effectiveness. Most evidence comes from retrospective, frequently single-center studies of diagnostic performance; few studies have measured effects on workflow, and fewer still on patient outcomes or cost. Routine clinical adoption will require prospective multicenter trials, standardized performance benchmarks, and evidence of clinical and economic benefit.

## 1. Introduction

Ischemic heart disease remains the primary contributor to global morbidity and mortality. According to the Global Burden of Disease study, there were an estimated 254 million prevalent cases and 9 million deaths attributable to this condition in 2021 [1]. Cardiovascular disease as a whole, chiefly ischemic heart disease and stroke, caused 18.6 million deaths in 2019 [2]. Myocardial ischemia spans a clinical continuum, from stable angina and silent ischemia to acute coronary syndrome (ACS). Identifying ischemia accurately and promptly across this spectrum is a core diagnostic challenge; each year, millions of adults are evaluated for chest pain in United States (US) emergency departments, yet only 5.1–8.4% are diagnosed with acute myocardial infarction (MI) [3]. Consequently, diagnostic pathways must identify all acute coronary occlusions while limiting unnecessary testing in the much larger group of patients without occlusion.

The diagnostic burden falls on three main sensing modalities, each with its own limitations. Although the electrocardiogram (ECG) is rapid and universally available, its conventional criteria for acute ischemia are highly specific but have low sensitivity [4]. A substantial proportion of acute coronary occlusions do not satisfy the established diagnostic criteria that have guided clinical triage for decades. This limitation has led to growing interest in an occlusion myocardial infarction (OMI) framework, which aims to identify patients with an acutely occluded coronary artery who need urgent reperfusion, whether or not ST-elevation criteria are met [5]. Cardiac troponin, a biochemical marker of myocyte injury, is highly sensitive, but its concentration can be low early after symptom onset, even with high-sensitivity assays [6], and varies with patient age, sex, and renal function [7]. Cardiac imaging enables direct visualization of myocardial structure and function, but it can be costly and involve ionizing radiation [8] or contrast agents that some patients cannot receive [9], and its interpretation depends on operator skill and experience [10,11].

Artificial intelligence (AI), particularly deep learning, is increasingly being applied across these diagnostic modalities. By directly identifying patterns from raw sensor data, these AI-enabled methods can detect latent features that are missed by traditional rule-based interpretation [12]. In this review, we organize each modality as a bioelectric, biochemical, or structural and functional sensor and examine how AI may improve the detection of myocardial ischemia and its related diagnostic targets. We also discuss foundation models, multimodal approaches, relevant datasets and performance measures, regulatory considerations, and the main barriers to clinical translation.

Cardiac ischemia is the organizing concept of this review, but the studies it covers do not all address the same diagnostic target. Six related endpoints recur: acute coronary occlusion; acute MI; myocardial injury, meaning troponin release without a specified mechanism; established or chronic MI; anatomical obstructive coronary artery disease (CAD) and plaque burden; and functional ischemia, shown by stress-induced perfusion or wall-motion abnormalities or by reduced fractional flow reserve (FFR). These endpoints are linked mechanistically but distinct diagnostically. A model that detects an angiographically confirmed acute occlusion is solving a different problem from one that estimates the probability of troponin-defined infarction, quantifies stenosis on computed tomography (CT), or identifies scar on magnetic resonance imaging, so performance figures are not interchangeable across these targets. Table 1 lists representative studies for each sensing modality and the endpoint each addressed.

## 2. Methods

This is a narrative review. PubMed/MEDLINE was searched from inception to 30 June 2026, combining AI terms (“artificial intelligence”, “machine learning”, “deep learning”, and “neural network”) with ischemia terms (“myocardial ischemia”, “acute coronary syndrome”, “myocardial infarction”, “coronary artery disease”, and “coronary occlusion”) and with modality-specific terms for each sensing domain (for example, “electrocardiography”, “troponin”, “echocardiography”, “computed tomography angiography”, “cardiac magnetic resonance”, and “myocardial perfusion imaging”). Additional searches covered foundation models, multimodal learning, datasets, and clinical translation. English-language studies applying AI to a cardiac sensing modality for the detection, characterization, or risk stratification of ischemic heart disease were eligible, as were relevant technical reports, reviews, study protocols, public dataset resources, and consensus and epidemiological sources. Selected studies outside ischemic heart disease were included to illustrate methodological or implementation issues and are identified as such in the text. Animal-only studies and conference abstracts were excluded. Reference lists of retrieved articles were screened for additional sources, and public dataset resources and articles in journals not indexed in MEDLINE were identified by targeted searching. Selection among eligible studies was purposive, favoring clinical relevance, external or multicenter validation, and coverage of the main sensing modalities, and findings were synthesized narratively.

## 3. The Electrocardiogram as a Bioelectric Sensor

The ECG is the most established cardiac sensor in routine clinical practice. Cutaneous electrodes capture cardiac bioelectric activity, while the standard 12-lead layout provides a multi-axial view of this electrical vector. Before analysis, signals are filtered to reduce baseline drift and muscle artifact. Ischemia primarily manifests as changes in the ST segment, the interval between ventricular depolarization and repolarization, with deviation from the isoelectric baseline indicating an injury current. Conventional interpretation compares this segment against fixed thresholds, historically defined by specific amplitudes of ST-segment elevation across contiguous leads [4,29]. Deep learning shifts this approach; rather than a few human-defined measurements, convolutional neural networks learn features directly from digitized waveforms, capturing distributed inter-lead and beat-to-beat patterns that conventional criteria often miss. The terms AI, machine learning, and deep learning are often used loosely. AI is the broadest category; machine learning is the subset of methods that learn parameters from data; and deep learning is the subset of machine learning that uses multilayered neural networks. We use each term at its own level of specificity. Performance depends on both signal quality and model design; parameters such as lead count, signal fidelity, and the accuracy of the training reference standard together set the ceiling on achievable accuracy. Phenotypic selectivity, meaning the specific conditions a model is actually able to discriminate, should also be assessed before clinical deployment [30]. A model may detect more than the condition it was built for. In a phenome-wide analysis, AI-enhanced ECG (AI-ECG) models built to detect left ventricular systolic dysfunction, aortic stenosis, mitral regurgitation, and left ventricular hypertrophy were each associated with their intended condition but showed similar or stronger associations with many other cardiovascular phenotypes [30]. Such a model may behave as a general marker of cardiovascular disease, which an area under the receiver-operating-characteristic curve (AUC) against its intended label will not reveal.

### 3.1. Acute Coronary Occlusion and Occlusion Myocardial Infarction

A primary diagnostic challenge in ACS involves acute coronary occlusions that do not produce ST-segment elevation. One-quarter to one-third of patients categorized as having non-ST-elevation myocardial infarction (NSTEMI) have an angiographically documented culprit occlusion; these patients often have delayed reperfusion and higher mortality than those without occlusion [5,15]. The ECG often contains the relevant information, but traditional, rule-based interpretation frequently fails to extract it; AI-ECG models are designed to close this gap.

In the prehospital setting, using clinician-adjudicated outcomes validated by angiography as a reference standard, Al-Zaiti and colleagues developed machine-learning models on 12-lead signals from 4026 patients presenting with chest pain, reaching an AUC of 0.91 on an internal hold-out test set for occlusion detection, exceeding both commercial software (0.78) and clinician interpretation (0.79). In the development data, a risk score derived from the model ruled out occlusion with 0.91 sensitivity and a 0.993 negative predictive value (NPV). In external validation at two independent sites (n = 3287), discrimination fell to 0.87, still exceeding clinicians (0.80) and commercial software (0.75), and the score placed 69% of patients in its low-risk group with a 1.3% false-negative rate [14]. Consistent with these data, an international model evaluated on 3254 tracings achieved 2.5 times the sensitivity of ST-elevation criteria (80.6% vs. 32.5%) at a modest cost in specificity (93.7% vs. 97.7%); at a threshold set to match the specificity of ST-elevation criteria, model sensitivity was 65.8%, still twice that of the criteria themselves [15]. In a retrospective analysis of 217 patients undergoing angiography, a commercially available model detected occlusion with 86.5% sensitivity and 82.2% specificity (AUC 0.84), compared with 54.1% sensitivity and 88.7% specificity with ST-elevation criteria. Model-identified occlusion was significantly associated with in-hospital mortality (odds ratio 12.44, 95% confidence interval (CI) 1.56–98.98), although the interval is very wide; the one-year outcomes assessed (reduced ejection fraction, unplanned readmission) did not differ, and all estimates were unadjusted [16].

A related development is the OMI framework, which has emerged as an alternative, mechanism-based approach that seeks to identify patients requiring urgent reperfusion beyond conventional ST-elevation criteria. OMI is a conceptual and clinical framework rather than a universally interchangeable replacement for ST-elevation myocardial infarction (STEMI) and NSTEMI terminology, which remains embedded in current guidelines, coding, and quality-reporting systems. The definition of occlusion also varies between studies, and the evidence for AI-based occlusion detection depends on it. The framework’s diagnostic value is evident even without automation: when an expert interpreter applied explicit OMI criteria instead of conventional ST-elevation thresholds, sensitivity for detecting occlusion rose from 41% to 86% while specificity fell slightly, from 94% to 91% [13]. Several of the models described above were already trained against adjudicated occlusion rather than ST-elevation criteria [14,15], and extending this approach more widely is a plausible next step for the field.

### 3.2. Recognition of ST-Elevation Myocardial Infarction and Catheterization Laboratory Activation

In interventional workflows, AI interpretation of the ECG, applied retrospectively, would have increased diagnostic sensitivity and reduced unnecessary catheterization laboratory activations [17,31], which would spare patients unnecessary invasive angiography. A review of AI in interventional cardiology cautions, however, that such tools should be adopted only where they offer a clear advantage over standard care [32]. In a retrospective analysis of a multicenter US registry of 1032 patients who had already triggered emergent catheterization laboratory activation at three percutaneous coronary intervention centers, AI interpretation of the ECG increased sensitivity from 71.0% to 92.0% and reduced false activations from 41.8% to 7.9% [17]. Specificity rose from 29.0% to 81.0%, the larger effect and the one that accounts for the fall in false activations. Because the algorithm was applied after the fact, these gains are modeled rather than observed, and the study does not establish the effect of real-time deployment. Similar findings have been reported before hospital arrival. In a single-center retrospective study of 117 patients for whom paramedics intended prehospital activation, 48 of whom had OMI, an AI algorithm reduced false-positive activations relative to paramedic judgment by 34 percentage points, a reduction not significantly different from the 27 points achieved by ST-elevation criteria; the algorithm missed no occlusions, whereas ST-elevation criteria missed six of the 48 [31]. Predictive models may also aid downstream decisions. For example, an algorithm trained on 144,691 emergency department presentations predicted the need for revascularization with an external-validation AUC of 0.81, outperforming clinician ECG interpretation (0.70), though not high-sensitivity troponin (0.85) [33].

These results warrant caution, as most studies are retrospective. Two further limitations are independent of study design. First, several of these evaluations use commercial algorithms whose internal logic is not disclosed, so reported performance cannot be attributed to any specific modeling choice. Second, as noted in Section 3.1, the definition of occlusion used to adjudicate the reference standard varies between studies, and the same model can appear more or less sensitive depending on which definition is used. The clinical implications of negative findings also matter. For instance, in patients with suspected high-risk non-ST-elevation ACS, a negative output from an AI-ECG model on the first ECG did not reliably exclude coronary occlusion (NPV 57%), and so cannot replace established serial testing [34]. Prospective evidence is beginning to appear. A cluster-randomized trial (ARISE) involving 43,234 participants reported a 99.9% NPV for AI-assisted STEMI detection, which the authors note may be overestimated because of pragmatic data collection [18]. This trial deserves particular weight because it is one of very few in this literature to measure clinical workflow prospectively rather than diagnostic accuracy retrospectively; it reported shorter door-to-balloon and ECG-to-balloon times. Its primary analysis, however, rested on 137 emergency department patients with STEMI at a single center, and time to treatment is a process measure. The trial was powered for door-to-balloon time, not clinical outcomes; cardiac death, a secondary endpoint, was lower in the intervention arm, but the authors caution that this may not reflect the AI alerts [18]. In addition, a multicenter registry has been designed to collect ECG data during angiography to benchmark performance against coronary flow [35].

### 3.3. Exercise Stress Testing: Functional Ischemia

Exercise ECG remains widely used in functional testing for CAD, though its sensitivity is limited, particularly in women [8]. Interpretation is further complicated by signal degradation, including baseline wander and muscle artifact, and by the subtlety of ST-segment changes relative to heart rate, which observers interpret differently. AI analyzes the full waveform rather than relying on ST-segment thresholds alone. For instance, a single-center model trained on 1200 exercise ECGs to predict obstructive CAD on invasive angiography achieved 93.6% sensitivity and 93.2% specificity in internal validation. However, accuracy declined to 78% in a separate validation cohort of 91 patients, underscoring the persistent challenge of generalizability [8]. The decline was driven by sensitivity, which fell from 93.6% to 64.6% while specificity was preserved at 93%; the model missed more disease without generating more false positives. Several contributors are plausible, and the study design cannot separate them. The validation cohort was small, so the estimate is imprecise; it was drawn from a later, partly overlapping period (2002–2021, against 1993–2010 for development); and a model developed on 1200 ECGs from one institution has limited opportunity to learn features that generalize. The result shows how little internal validation reveals about performance elsewhere. In detecting functionally significant disease, models modestly improve on standard stress ECG [36]. These algorithms may also improve triage. An analysis of 2325 treadmill examinations found that an algorithmic model maintained diagnostic sensitivity while reducing the false-positive rate from 76% to 55% [37], indicating potential to decrease unnecessary invasive angiographic procedures. Despite these advances, much of the supporting evidence comes from retrospective, single-center studies.

### 3.4. Wearable and Ambulatory Monitoring: ST-Segment Change

Wearable and ambulatory monitoring platforms expand ECG-based diagnostics beyond the clinic, but at the cost of fewer leads and lower signal quality. Single-lead recordings are often insufficient to diagnose infarction [38], and wearable recordings are prone to noise from poor electrode contact and movement [39]. Direct evidence for ischemia detection is correspondingly weak, and the endpoint varies: some studies report detection of perfusion-defined ischemia, others ST-segment change, and others STEMI. During pharmacological stress, ST-segment deviation recorded by a wireless single-lead system identified reversible anterolateral ischemia with a sensitivity of only 8.3%, and 12-lead ECG performed little better in the same cohort, at 12.5% [40]. Both estimates rest on only 24 patients with ischemia and have very wide CIs (1.0–27.0% and 3.0–32.4%, respectively); they show that single-lead sensitivity is poor, but its true value is uncertain. Despite these limits, such devices could in principle help detect silent ischemia and shorten delays to presentation, although neither benefit has yet been shown. Recent studies show feasibility; for instance, one using a consumer smartwatch repositioned to simulate precordial leads reported 93% sensitivity and 95% specificity for ST elevation against a simultaneous 12-lead ECG, in patients with known MI and healthy controls [41], while a deep-learning model identified STEMI with 90.5% accuracy from lead V2 alone, extracted from standard 12-lead ECGs [42]. Self-supervised pre-training on a wearable corpus of 658,486 recordings, of which only 164,538 were annotated, has also enabled wearable 12-lead models; the same approach underlies the foundation models discussed in Section 6 [43]. However, these numbers need scrutiny. A scoping review found that reported accuracy rates, often above 99%, dropped in several studies when subjected to rigorous inter-patient and external validation [44]. The evidence for wearable ST-segment ischemia detection therefore remains preliminary, well behind the established use of these devices for arrhythmia monitoring.

## 4. Cardiac Biomarkers as Biochemical Sensors

Cardiac troponin is the primary biochemical reference standard for myocardial injury. As a structural protein released into the blood during cardiomyocyte damage, it acts as a sensitive sensor; assays are now sensitive enough to detect troponin in most healthy individuals. This heightened sensitivity, however, creates a clinical paradox: although it improves the detection of genuine infarction, raised concentrations in older patients and in those with renal dysfunction or non-coronary conditions reduce specificity when read against a single fixed threshold. Machine learning methods address this tension by preserving the analytical sensitivity of modern assays while replacing static thresholds with individualized estimates of infarction probability. Troponin reflects myocardial injury, not ischemia. Models built on troponin therefore estimate the probability of infarction, or of a particular type of injury, and their performance is not evidence of ischemia detection as such.

One such model, the Myocardial-Ischemic-Injury-Index, a gradient-boosted algorithm incorporating age, sex, and serial high-sensitivity troponin concentrations, provides a continuous probability estimate rather than a binary result, and was trained on 3013 patients and tested on 7998 [19]. The CoDE-ACS model extended the approach to a single troponin measurement combined with clinical features; validated across seven external cohorts involving over 10,000 patients, it reached an AUC of 0.95 and triaged a substantially larger proportion of patients as low-risk at presentation than fixed thresholds, with a similar NPV [7]. When benchmarked against guideline-recommended pathways requiring 0-, 1-, and 2-h sampling, CoDE-ACS ruled out more patients at presentation than both the European Society of Cardiology (ESC) 0-h and High-STEACS protocols, while sustaining an NPV of 99.7% [45]. Models can also distinguish type 1 infarction (from plaque rupture or erosion) from type 2 infarction (from supply–demand mismatch) or acute non-ischemic myocardial injury with reported AUCs of 0.955 and 0.946 for gradient-boosted and deep-learning models, respectively [46].

These methods also extend to the point of care. For example, when applied to a single point-of-care high-sensitivity troponin measurement in 2560 patients, a machine-learning algorithm identified 35.1% as suitable for rapid rule-out, with an NPV of 99.96%, more than twice the proportion identified by the ESC and American College of Cardiology 0-h pathways (15.2% and 13.8%) [20]. Point-of-care assays return results faster than laboratory assays, but whether this shortens emergency department stays is unproven [20]. Pooled evidence is also encouraging: a meta-analysis of nine studies and 6292 patients evaluating machine-learning approaches to ACS reported a pooled sensitivity of 0.95 and a specificity of 0.90, although the pooled studies were heterogeneous and, as the authors note, may not apply fully to current high-sensitivity troponin assays [47]. Even so, we see these models as ready for implementation studies in specific settings, not for immediate, unrestricted clinical use. Most of these models have been developed and validated using European and Australasian cohorts, with some North American validation [7,19,20]. Troponin models are also limited by the reference standards against which they are judged [48].

Because troponin concentrations can be low soon after symptom onset, some models add biomarkers that rise earlier. For example, a machine-learning algorithm applied to a multiplexed immunoassay of copeptin, cardiac troponin I, and heart-type fatty-acid-binding protein outperformed troponin alone in the early hours after symptom onset [6]. However, in this case and many others, small-scale studies with near-perfect discrimination should be viewed as proofs of concept rather than definitive evidence. Combinations of circulating biomarkers and imaging are at a similar stage. In 1069 asymptomatic adults, a machine-learning score combining serum biomarkers with clinical data and CT measures, including the coronary artery calcium score, predicted MI or cardiac death over a mean of 14.5 years better than the calcium score alone (AUC 0.81 vs. 0.75), although it was assessed only by cross-validation (CV) within that cohort [49]. Models that integrate the ECG with routine blood tests at presentation have ruled out MI or death within 30 days in more than half of patients with chest pain, with a sensitivity above 99%, in a retrospective two-center analysis [50]. Integrated, individualized outputs of this kind are promising but are best treated as complementary to other sources of information.

## 5. Cardiac Imaging as a Sensing Modality

Each cardiac imaging technique detects ischemia, or its anatomical substrate, through a different physical signal: backscattered ultrasound in echocardiography, X-ray attenuation in CT, tissue magnetization in cardiac magnetic resonance (CMR), and photons emitted by radiotracers in nuclear imaging. AI contributes in three ways. The first is automation: measurements that once required manual tracing are produced directly from the image. The second is feature extraction: models learn image characteristics that conventional measurements do not capture and that a reader cannot easily quantify. The third is prediction: those features are used to predict ischemia and downstream clinical risk.

Two points apply across all four modalities. First, the endpoints differ: a model detecting regional wall-motion abnormality (RWMA) addresses myocardial dysfunction, one quantifying stenosis on coronary CT angiography (CCTA) addresses anatomical obstruction, and one predicting a perfusion deficit addresses functional ischemia. These endpoints are related but not interchangeable, and performance figures should not be pooled across them. Second, the reference standard varies within each modality, from invasive quantitative coronary angiography (QCA) in some studies to expert-reader consensus or clinical report text in others, and the choice materially affects reported accuracy.

### 5.1. Echocardiography

RWMA is the clearest echocardiographic sign of ischemia and also the most subjective. Convolutional neural networks, which learn spatial features directly from image sequences, suit this task because the diagnostic information lies in myocardial motion across the cardiac cycle rather than in any single frame. In patients with prior MI, a convolutional neural network assessing regional wall motion matched the performance of expert cardiologists and sonographers (AUC 0.99 vs. 0.98) while significantly outperforming resident readers (0.90) and distinguishing among coronary infarction territories [10]. A separate network, developed on routine clinical workloads at one tertiary center and externally validated at a second, identified RWMAs, with an AUC of 0.912 in CV and 0.891 in external validation [51].

The most direct reader comparison comes from a model that predicts RWMA in seven regions defined by coronary perfusion territories, developed on 15,746 transthoracic studies (14,072 for training) comprising 25,529 apical videos [52]. Against a test-set ground truth defined by expert readers, it detected any abnormality with an AUC of 0.96 (95% CI 0.92–0.98). Its F1 scores were numerically similar to those of six experts in six of seven regions and lower only in the anteroseptal region (75 vs. 89), and it outperformed two of three novice readers in detecting any abnormality. The comparison is useful because it shows where the model is weakest, not only how it performs overall.

In a randomized crossover reader study, an automated classification of stress echocardiograms increased clinicians’ sensitivity for severe CAD by 10%, as well as their agreement and confidence [21]. In 512 participants across seven hospitals in the United Kingdom (UK) and US, AI-derived left ventricular ejection fraction and global longitudinal strain at peak stress achieved AUCs of 0.86 and 0.82, respectively, for angiographic CAD, compared with 0.92 for visual wall-motion score index, and adding either measure to wall-motion scoring significantly improved discrimination [53]. The study shares a limitation common in stress echocardiography research: only 24% of participants underwent angiography, so the reference standard was applied selectively. In a multicenter evaluation of automated chamber quantification during pharmacological stress echocardiography (240 patients, five centers), agreement with expert cardiologists was strong for left ventricular volumes (correlation 0.92–0.95 in apical four-chamber and 0.89–0.91 in two-chamber views), but measurement yield was lower for right-sided and atrial chambers (75–83%) [11]; automation is therefore not uniformly reliable across the measurements a stress study generates.

### 5.2. Coronary CT Angiography

CCTA has been a particular focus of AI development, largely because manual measurement of plaque and stenosis in three dimensions is labor-intensive and operator dependent. AI has been used primarily in two ways. First, when assessing anatomy, AI-based stenosis quantification on CCTA has shown substantial agreement with expert core-laboratory QCA [54], while automated plaque quantification in multicenter cohorts has shown independent prognostic value [22]. In a multisite study of 232 patients, with the consensus of three level-3 readers as ground truth, an analysis service cleared by the US Food and Drug Administration (FDA) agreed within one Coronary Artery Disease Reporting and Data System (CAD-RADS) category in 98.3% of patients and 99.9% of vessels [55].

The most rigorous reader comparison used an invasive reference standard rather than expert consensus. Among 208 patients with suspected CAD, AI-based quantitative CT detected obstructive stenosis on invasive QCA with a per-patient AUC of 0.91, outperforming one level-3 reader (0.77) and two level-2 readers (0.79 and 0.76; all *p* < 0.002) [56]. Per vessel, the model performed similarly to the level-3 reader (0.86 vs. 0.82, *p* = 0.098) and remained superior to both level-2 readers (0.69 each, *p* < 0.001). Its advantage over the level-3 reader was confined to patients with above-median plaque volume, where, the authors suggest, reader variability matters most; below the median, the two did not differ [56]. In this single-center analysis, AI matched or exceeded an experienced level-3 reader and outperformed less experienced readers.

The second use is physiological assessment. CT-derived FFR estimates the hemodynamic significance of lesions seen on CCTA. In a meta-analysis of 16 studies, the pooled per-patient estimates (seven studies, 1013 patients) were a sensitivity of 89% and a specificity of 71% [57]. The gain over anatomical assessment lies in specificity: in the same analysis, CCTA alone had a comparable per-patient sensitivity of 93% but a specificity of only 32%. CT-derived FFR is therefore better at excluding ischemia than at confirming it, and its residual false-positive rate, rather than missed disease, is the main constraint on its use as a gatekeeper. Newer models predict vessel-specific ischemia by analyzing plaque characteristics together with vessel morphology. In a post hoc analysis of two prospective studies, one such model identified ischemia, defined by invasive FFR, more accurately than CT-derived FFR in external validation (per-patient AUC 0.85 vs. 0.78) and comparably to positron emission tomography (PET; 0.89); a positive result was also associated with major adverse cardiovascular events independently of stenosis, although with a wide CI [23].

### 5.3. Cardiac Magnetic Resonance

CMR is widely used for myocardial tissue characterization, and AI is being applied across its workflow, from acquisition to interpretation, with the largest impact so far on image segmentation and analysis [58]. Two uses in ischemic heart disease stand out. First, AI can detect infarction without gadolinium contrast; one model identified chronic infarction from non-contrast cine imaging with a per-segment AUC of 0.94, yielding infarct sizing results comparable to late gadolinium enhancement [9]. Because gadolinium-based contrast is often avoided in severe renal impairment, this would be valuable if confirmed, but the study was a retrospective, single-vendor proof of concept in which every patient also underwent contrast imaging. Second, AI is being developed to automate the laborious post-processing that quantitative stress-perfusion CMR requires, although robust segmentation across multicenter datasets has so far been demonstrated only in proof-of-concept work [59].

Here the constraint is operational rather than diagnostic. Conventional motion correction for quantitative perfusion relies on iterative image registration, which is computationally intensive and sensitive to changes in acquisition. An unsupervised deep-learning pipeline, developed on multivendor data from 201 patients (149 for training) and tested on 38 held-out cases from a single scanner, achieved myocardial alignment comparable to the established method (Dice 0.92 vs. 0.91, both improved from 0.80 before registration) and cut processing time about 15-fold for a representative case [60]. Such gains have not been shown to improve diagnostic accuracy, but by easing post-processing they could, as the authors suggest, support wider clinical use of quantitative perfusion CMR. As with other imaging modalities, however, many of these models remain limited by single-vendor, single-center development.

### 5.4. Nuclear Myocardial Perfusion Imaging

Because myocardial perfusion imaging is routinely quantified, it suits machine learning well. Deep learning predicts obstructive disease from single-photon emission computed tomography (SPECT) perfusion images more accurately than the standard total perfusion deficit [24]. An interpretable model trained on a large cohort outperformed expert readers [25], and in a later prospective study, physicians who read with its output were more accurate than without it [61]. In PET, a model combining perfusion, flow, and coronary calcium in 1664 patients drawn from a 17,348-patient multicenter registry outperformed experienced clinicians [26], although by a narrow margin (AUC 0.83 vs. 0.80, *p* = 0.02), and was externally validated at three independent sites.

Two modality-specific problems show where AI helps most. The first is attenuation artifact, the main source of false-positive SPECT results; CT-based attenuation correction is unavailable on most installed systems, adds radiation, and is degraded by misregistration. A conditional generative adversarial network trained at one site (n = 4886) and externally validated at two others (n = 604) generated simulated attenuation-corrected images from uncorrected data in under one second, raising the AUC for obstructive CAD from 0.70 (95% CI 0.63–0.78) to 0.79 (0.72–0.85), not significantly different from true attenuation correction (0.81, 0.75–0.87; *p* = 0.196) [62]. The second is interpretation of stress-only studies, which shorten scanning and reduce radiation exposure. In 37,243 patients from a single center, a deep convolutional neural network applied to stress-only images separated normal from abnormal perfusion, as judged by clinical nuclear cardiologists, better than conventional defect-size quantification (AUC 0.872 vs. 0.838, *p* < 0.01). At each method’s optimal threshold, the network had higher specificity (85.6% vs. 77.7%) but lower sensitivity (75.0% vs. 80.0%); at matched specificity, the two had similar sensitivity (81.2% vs. 80.0%) [63].

### 5.5. Reader Efficiency and What Remains Unproven

Across modalities, the most consistently demonstrated benefit is to the reader rather than the patient. In a reader study of 240 patients, physician interpretation supported by an explainable deep-learning prediction achieved a higher AUC than unaided interpretation (0.779 vs. 0.747, *p* = 0.003), with a net reclassification improvement of 17.2% (95% CI 9.2–24.4%) [61], although the benefit varied between readers, reflecting how willing each was to act on the model output. Several of the studies above report faster processing, such as sub-second attenuation correction [62] and quicker perfusion post-processing [60], but none measured the effect on reporting time or workflow.

What is largely missing is prospective evidence that these gains change patient management or outcome. The imaging literature is predominantly retrospective, and prospective trials of AI-guided management are rare. The principal exception is a randomized, multicenter, non-inferiority trial of AI-assisted stress echocardiography (PROTEUS) [64], which randomly assigned 2341 patients with suspected CAD at 20 UK National Health Service hospitals; 2213 completed six-month follow-up [65]. Its results are instructive because they are mixed. The primary endpoint, appropriate referral for invasive angiography, was analyzed as an AUC: 0.63 with AI and 0.55 without (difference 0.09; 95% CI −0.22 to 0.39), which did not meet the prespecified non-inferiority margin of −0.05 [65]. The point estimate favored AI, but only 85 participants were referred for angiography, and the authors acknowledge that the trial was underpowered to show non-inferiority [65]. Neither quality-adjusted life-years (0.392 intervention vs. 0.390 control, *p* = 0.818) nor angina scores differed, and the embedded economic evaluation found an incremental cost-effectiveness ratio of £6939 per quality-adjusted life-year before AI costs; the intervention remained cost-effective at an AI cost of up to £35.35 per case, or £45.93 when clinician time savings, estimated from outside the trial, were included [66]. A parallel mixed-methods evaluation of implementation barriers and acceptability accompanies the trial [67]. Until trials designed around patient outcomes are reported, claims for AI in cardiac imaging should be confined to diagnostic performance and reader-level effects.

## 6. Foundation Models and Multimodal AI

The first generation of AI-enabled cardiovascular sensors relied on task-specific development and validation: a model learns a representation of the input signal and its association with a single intended endpoint, and transfers poorly beyond it. Foundation models take the opposite approach. Borrowed from advances in computer vision and natural language processing, they are pre-trained on large unlabeled datasets by self-supervised learning and then adapted to many downstream tasks with little additional annotation [68]. The distinction lies in how a model is trained and reused rather than in its architecture: describing a network as a transformer, or as AI-ECG, does not make it a foundation model. At present, cardiac foundation models are at an early stage and lag behind those in general vision and language.

A prominent example is ECG-FM, an open-access ECG foundation model pre-trained on about 1.4 million ECG segments; after fine-tuning, it outperformed task-specific baselines when data were limited [27]. Its main advantage is label efficiency, which addresses the shortage of expert annotations. Unlike the task-specific AI-ECG models discussed earlier, each trained for a single labeled endpoint, ECG-FM is pre-trained on unlabeled recordings and then fine-tuned for each downstream task, so one pre-trained model can serve several endpoints [27]. The underlying method, self-supervised learning, extracts reusable representations from unlabeled ECGs [69] and extends to imaging; for instance, a self-supervised echocardiographic model was accurate for severe aortic stenosis despite limited labeled data [70]. Vision transformers have also been applied in cardiac imaging, for example to coronary vessel and plaque segmentation and disease detection on CCTA [71].

A further prospect is multimodal fusion, which integrates the bioelectric, biochemical, and imaging data streams examined separately above; early examples combine the ECG with routine blood tests [50] and circulating biomarkers with CT measures [49]. AI is also being paired with new sensors. In a retrospective single-center study of 2118 participants, a lightweight model designed for edge devices classified ischemia from single-channel magnetocardiographic recordings made with a new room-temperature magnetometer, although it has yet to be tested on the device itself [28]. Large language models (LLMs) are a separate track; general-purpose models often match or beat specialized clinical systems on medical benchmarks [72]. In a multicenter study of CMR reports, GPT-4 diagnosed myocarditis about as accurately as a first-year radiology resident but less accurately than more experienced residents [73]. However, these models work mainly with text, which is different from detecting ischemia directly from physiological signals. While foundation models are a notable methodological development, to our knowledge no cardiac foundation model—as distinct from task-specific AI-ECG models—has yet been validated as a diagnostic device, and a review of foundation models built on health-record data found that broad claims about them often rest on inconsistent evaluation [74].

## 7. Datasets, Benchmarks, Translation, and Open Challenges

### 7.1. The Reference Standard Problem in Cardiovascular AI

A supervised model can be no better than the labels it learns from, and across the modalities reviewed here the label is not a single, agreed quantity. Ground truth differs between modalities, between studies within a modality, and sometimes between analyses of the same dataset. The choice determines what a reported sensitivity or AUC actually means, and it is the main reason performance figures should not be pooled across studies without first confirming that they were measured against the same standard.

For the ECG, a model described as detecting occlusion may have been trained against any of several standards: an ST-elevation diagnosis recorded at presentation, angiographically documented coronary occlusion, a Thrombolysis in Myocardial Infarction (TIMI) flow grade threshold, a culprit lesion accompanied by troponin release, or clinician adjudication of the final diagnosis. Even studies that share the occlusion concept define it differently. Al-Zaiti and colleagues counted a culprit artery with TIMI flow grade 0–1, adjudicated by independent reviewers, or grade 2 with severe stenosis and high troponin [14]; the international evaluation also counted culprit lesions with TIMI grade 2–3 flow that required emergent or urgent revascularization [15]. That study found comparable performance across alternative definitions [15], but such robustness must be shown for each model rather than assumed, and AI-ECG studies are best compared when their adjudication standards match.

For cardiac biomarkers, the question is which infarction is being predicted. Adjudicated type 1 MI, any MI, NSTEMI, and adjudicated ACS are different labels with different prevalences, and a model trained on one will not transfer cleanly to another. Models that separate type 1 from type 2 infarction make the label explicit [46]; most do not. Incorporation bias is a related problem. In CoDE-ACS, for example, clinicians adjudicated the reference diagnosis of MI using features that the model itself takes as inputs, such as chest pain and ischemic ECG changes, so agreement with the reference standard is partly built in [48].

In imaging, the same problem appears as a choice between anatomical, physiological, and expert reference standards. CCTA models have been validated against invasive QCA [56], expert-reader consensus [55], and invasive FFR [23]. These standards do not measure the same thing, and a model that agrees closely with expert readers may agree less well with an invasive standard. In echocardiography, ground truth for RWMA has come from both clinical report text and dedicated expert re-reading [52], and the former carries whatever variability the original reporting contained. In CMR, late gadolinium enhancement is the reference for infarction [9], so a non-contrast model’s performance measures agreement with an existing imaging standard rather than with tissue pathology.

The consequences can be large, as an example from outside ischemic heart disease shows. In the amyloidosis-screening analysis discussed below, stricter case definitions raised the AUC on matched test sets from 0.705 to 0.880, but in the general patient population the models performed similarly (0.702 to 0.728) [75]; accuracy against a curated label did not carry over to the population in which screening would take place. The reference standard should therefore be reported alongside every performance metric, comparisons across studies should be limited to those that used the same reference standard, and regulatory and guideline evaluations of cardiovascular AI should require the reference standard to be specified for each claim.

### 7.2. Datasets and Benchmarks

The findings in this review depend on the data used for model development and evaluation, and progress has closely tracked the release of large, well-curated datasets. For ECG analysis, prominent examples include the Brazilian CODE cohort [76], MIMIC-IV-ECG [77], and the PhysioNet/Computing in Cardiology challenge datasets [78]. For imaging, the ACDC [79] and M&Ms [80] CMR segmentation challenges and the EchoNet echocardiographic video repository, which enabled automated ejection-fraction assessment [81], serve as foundational benchmarks. Results on these benchmarks, however, need careful reading, for two reasons. First, as Section 7.1 sets out, reported accuracy is limited by the reference standard. For instance, an analysis of amyloidosis-screening models developed in a cohort of approximately 1.3 million ECGs from 341,989 patients (an example from outside ischemic heart disease, cited for its methodological point) found that accuracy varied widely with the case definition used and dropped markedly in a general population, showing that a high AUC on a curated test set can mislead [75]. This matters especially for models that screen for undiagnosed disease, where the case definition can change apparent performance substantially. Second, representativeness also matters, as many datasets come from single-center cohorts with limited demographic diversity, which worsens the bias and generalization problems addressed below.

### 7.3. Generalization, Drift, and Bias

Several remaining barriers to deployment are technical. Foremost is generalization; external validation often shows performance drops, as seen in an AI-ECG model for low ejection fraction tested at new sites, cited as a methodological example rather than as evidence on ischemia [82]. A systematic assessment of 486 AI models for cardiovascular risk prediction reported across 79 studies found a universal lack of independent external validation, with every model at high risk of bias on the Prediction model Risk Of Bias Assessment Tool (PROBAST) [83]. Post-deployment performance degradation, caused by temporal shifts in data distributions, has prompted work on drift detection and continuous model updating, illustrated here by an in-hospital mortality model for general medicine wards [84]. Dataset bias compounds this, as models trained on data that underrepresent some subpopulations often perform worse for those groups [85]. Federated learning may reduce the need to share patient data across institutions. In this decentralized approach, the model is trained locally at each institution, so data need not be transferred to a central repository. This method has progressed from theoretical studies to use in multi-institutional cardiac imaging research [86]. Interpretability also supports clinical trust and, where possible, may be better built into model design than added afterward [87].

### 7.4. Regulation and Health Economics

Regulation and health economics lag behind the technology. Many AI-based medical devices have been cleared in the US and Europe, but approvals to 2020 were concentrated in radiology and rarely carried high-risk designations; an analysis of approvals between 2015 and 2020 also found no regulatory pathway specific to AI- or machine-learning-based devices in either jurisdiction [88]. Economic evidence is still limited; nevertheless, a health-economic analysis in a pragmatic trial of an AI-ECG alert for high mortality risk, rather than for ischemia, concluded that the alert system was likely to be cost-effective in Taiwan, judged against a threshold derived from Taiwan’s willingness to pay per quality-adjusted life-year, since no accepted threshold per death averted exists [89]. Among 15,965 patients, 90-day all-cause mortality was 3.6% with the AI alert system versus 4.3% in controls, at an incremental cost of $59,500 per death averted (95% CI −$4657 to $385,950); the probability of cost-effectiveness reached 95% at a willingness to pay of $409,321 per death averted, just above the authors’ own acceptable range of $232,638 to $398,808. The point estimate is favorable, but the interval is wide and the conclusion tentative. We argue that the main barrier to the widespread clinical use of cardiac AI is no longer algorithmic performance alone, but the limited quality of the supporting evidence and the data used to develop these models. Realizing clinical benefit will therefore depend less on further model development than on the practical work of integration into workflows, governance, and monitoring at scale [90]. This aligns with a Lancet Digital Health series paper on cardiac imaging highlighting the continued lack of robust evidence that AI offers clear clinical or economic advantages over clinicians [91]. Prioritizing prospective, multicenter validation and the development of representative, high-quality datasets is likely to advance the field more than further architectural tuning.

### 7.5. From Diagnostic Performance to Clinical Utility

Studies in this field often report good discrimination as if it guaranteed clinical benefit. The two are different: a model can reach an AUC of 0.95 without changing clinical decisions, speeding up care, or improving outcomes. It is therefore useful to separate five levels at which an AI-enabled cardiac sensor can be evaluated and to state which level a given study reaches.

The first level is technical performance: AUC, sensitivity, and specificity against a stated reference standard, usually measured retrospectively and often in the development cohort. The second is diagnostic performance compared with the clinicians or conventional criteria the model is intended to assist or replace, as when AI-ECG models were compared with commercial software and clinician interpretation [14], AI-based quantitative CT with level-2 and level-3 readers [56], or interpretable PET models with experienced physicians [26]. The third is effect on clinical workflow, such as time to interpretation, catheterization laboratory activation, door-to-balloon time, and reader efficiency and confidence. The fourth is effect on patient outcomes: mortality, reinfarction, major adverse cardiovascular events, heart failure, bleeding, and hospitalization. The fifth is health-economic evaluation of cost-effectiveness and resource use.

**Figure 1:**
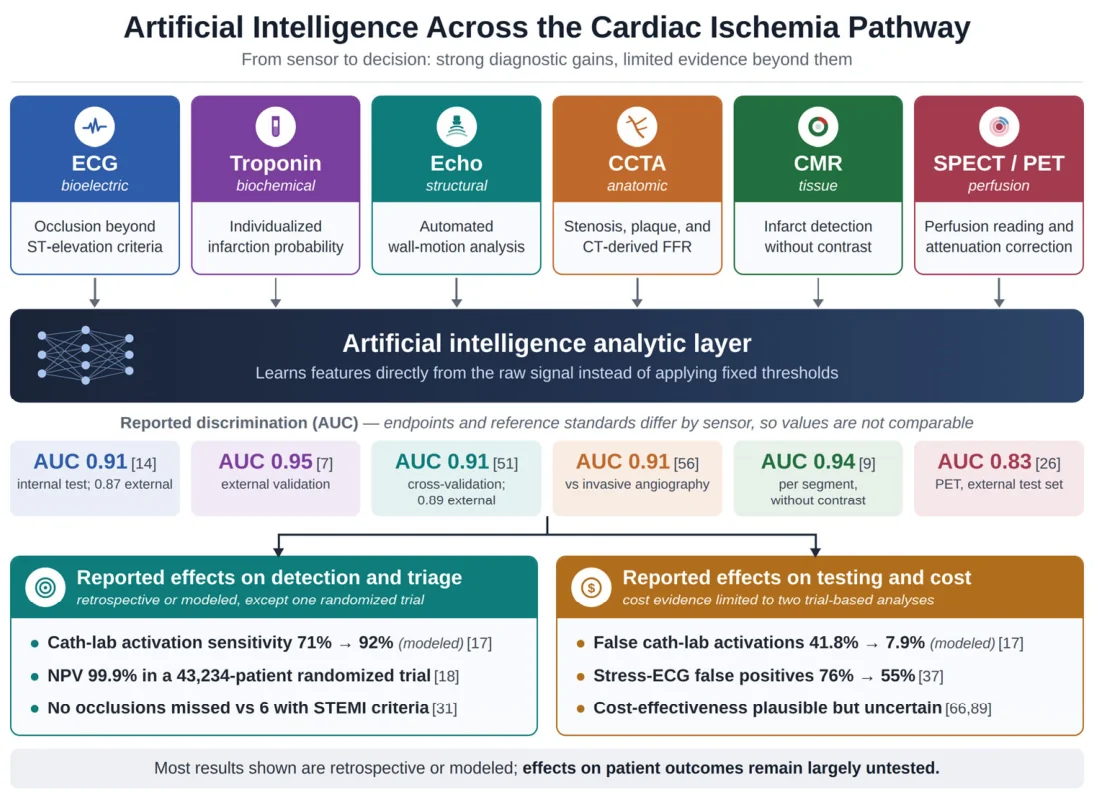
Artificial intelligence across the cardiac ischemia diagnostic pathway. AI acts as an analytic layer applied to established cardiac sensors: the ECG (bioelectric), cardiac troponin (biochemical), echocardiography (structural), CCTA (anatomic), CMR (tissue), and SPECT or PET myocardial perfusion imaging (perfusion). For each sensor, models learn features directly from the raw signal rather than applying fixed rule-based thresholds. The endpoint differs by sensor, so the AUCs shown are not comparable across sensors. The gain shown is in discrimination; whether it improves patient outcomes or resource use remains largely untested. Bracketed numbers denote the corresponding references. AI = artificial intelligence; AUC = area under the receiver-operating-characteristic curve; cath-lab = catheterization laboratory; CCTA = coronary computed tomography angiography; CMR = cardiac magnetic resonance; CT = computed tomography; ECG = electrocardiogram; FFR = fractional flow reserve; NPV = negative predictive value; PET = positron emission tomography; SPECT = single-photon emission computed tomography; STEMI = ST-elevation myocardial infarction.

The evidence reviewed here is unevenly distributed across these levels (Figure 1). The first two levels are well covered in all three sensing modalities. The third rests on a small number of studies, of which the cluster-randomized ARISE trial is the most substantial [18]; reader studies in nuclear imaging [61] and faster image processing [60,62] point toward workflow gains without having measured them. The fourth is sparse. The association between model-identified occlusion and in-hospital mortality is observational rather than interventional [16]; the ARISE trial reported fewer cardiac deaths as a secondary endpoint, which its authors caution may not reflect the alerts themselves [18]; and a pragmatic randomized trial of an AI-ECG alert for high mortality risk, rather than for ischemia, reported a 17% relative reduction in 90-day all-cause mortality (hazard ratio 0.83, 95% CI 0.70–0.99) [92]. The fifth consists of two health-economic analyses nested in randomized trials [66,89]. The stress echocardiography trial shows why the levels must be kept apart: AI-assisted reading did not meet its prespecified non-inferiority endpoint for appropriate referral to angiography [65] and made no difference to quality-adjusted life-years, yet was judged plausibly cost-effective, depending on the price of the AI and on clinician time savings estimated outside the trial [66]. A favorable result at one level does not establish the others.

The five-level framework also sets out what clinical translation requires, and in what order: technical validation; clinical validation against an appropriate comparator and reference standard; implementation and workflow evaluation in the setting of intended use; and outcome and health-economic assessment. Most cardiovascular AI has been evaluated only at the first two stages and has entered the third in isolated cases. Claims for this technology should not go beyond the highest level at which supporting evidence exists. Reporting a level-one result while describing a level-four benefit is, in our view, the most common overstatement in the field.

## 8. Limitations

This review has limitations. It is narrative rather than systematic: no protocol was registered, selection among eligible studies was purposive rather than exhaustive, and no formal risk-of-bias appraisal was undertaken. We pooled no results, so apart from two published meta-analyses [47,57], the figures quoted describe individual studies rather than summary estimates. Selection favored clinical relevance, external or multicenter validation, and coverage across sensing modalities, which may underrepresent smaller studies and those with negative findings. A few studies outside ischemic heart disease are cited for methodological points and are identified as such. The field is also moving quickly; our search closed on 30 June 2026, and both the models and the evidence for them will have moved on. For that reason, we have emphasized the level of evidence behind each claim, which changes more slowly than any individual performance figure.

## 9. Conclusions

Across several cardiac sensing modalities, AI has demonstrated improved discrimination compared with selected conventional rule-based approaches, particularly in retrospective and externally validated studies. Examples include detecting occlusions beyond ST-elevation criteria on the ECG, generating individualized risk from troponin, and improving quantification of stenosis, wall motion, and perfusion on imaging. However, evidence that these improvements consistently translate into superior clinical outcomes compared with contemporary guideline-based diagnostic pathways remains limited. Recent work has turned to foundation models and multimodal fusion, which aim to combine diverse inputs into patient-specific estimates of the endpoint in question, whether that is coronary occlusion, infarction, obstructive disease, or functional ischemia.

Wider clinical adoption is now limited less by the technology than by the evidence and infrastructure around it. The evidence base still consists largely of retrospective, single-center cohorts. External validation studies frequently show reduced performance, while model drift and entrenched biases continue to limit equitable care. Although prospective, randomized studies are beginning to appear, they remain uncommon. Economic and regulatory frameworks have not kept pace. The principal barriers are no longer the sensing modalities themselves; instead, the main challenges are weak evidence for model performance outside development settings and the lack of secure, standardized protocols for clinical integration.

Clinical use requires rigorous prospective validation against clinically meaningful endpoints, not benchmark accuracy alone. It also requires transparency about training datasets, reference standards, and performance stratified by patient subgroup. Integration into clinical workflows and guidelines is essential to build trust and ensure auditability. These are the standards expected of any diagnostic test, and new analytical methods should be held to them.

## Figures and Tables

**Table 1:** Representative studies of AI for cardiac ischemia detection, by sensing modality. The AI task column gives the endpoint each study actually measured; these endpoints are not interchangeable. ACC = American College of Cardiology; AI = artificial intelligence; AUC = area under the receiver-operating-characteristic curve; CAD = coronary artery disease; CCTA = coronary computed tomography angiography; CMR = cardiac magnetic resonance; cTnI = cardiac troponin I; CV = cross-validation; ECG = electrocardiogram; ESC = European Society of Cardiology; EU = Europe; FFR = fractional flow reserve; H-FABP = heart-type fatty-acid-binding protein; IVUS = intravascular ultrasound; MI = myocardial infarction; MPI = myocardial perfusion imaging; NPV = negative predictive value; OMI = occlusion myocardial infarction (see Section 3.1); PCI = percutaneous coronary intervention; PET = positron emission tomography; RCT = randomized controlled trial; SPECT = single-photon emission computed tomography; STEMI = ST-elevation myocardial infarction; UK = United Kingdom; US = United States.

Study	Modality/Sensor	AI Task/Endpoint	Cohort/Design/Validation	Key Performance
Meyers 2021 [13]	ECG (human OMI criteria)	Occlusion concept—acute coronary occlusion	808—Retrospective case–control; internal	OMI findings sensitivity 86% vs. 41% for STEMI criteria
Al-Zaiti 2023 [14]	ECG (prehospital)	OMI detection—acute coronary occlusion	7313—Observational cohort, multicenter; external test set (n = 3287)	AUC 0.91 (internal hold-out; 0.87 external); >clinicians (0.79) and software (0.78)
Herman 2024 [15]	ECG (12-lead)	OMI detection—acute coronary occlusion	3254 ECGs—Retrospective; internal EU test set (n = 2016) and external US test set (n = 1238)	Sensitivity 80.6% vs. 32.5% for STEMI criteria, specificity 93.7% vs. 97.7%
Choi 2026 (AERO-ACS) [16]	ECG (PMcardio)	OMI detection—acute coronary occlusion	217—Retrospective, single-center; external (independent validation of a commercial model)	Sensitivity 86.5% vs. 54.1%; higher in-hospital mortality if AI-positive
Herman 2026 [17]	ECG	Reduce false activations—acute coronary occlusion/catheterization laboratory triage	1032—Retrospective analysis of a prospective registry, 3 PCI centers; external	Sensitivity 71% → 92%; false activations 41.8% → 7.9%
Lin 2024 (ARISE) [18]	ECG	STEMI triage (RCT)—acute coronary occlusion/workflow	43,234—Cluster-randomized trial; prospective	NPV 99.9%; shorter time to treatment
Than 2019 (MI^3^) [19]	Troponin	Individualized MI probability—acute MI	>10,000—Analysis of prospectively collected data; trained 3013, held-out test set 7998	Probability score vs. fixed threshold; AUC 0.963 (test set)
Doudesis 2023 (CoDE-ACS) [7]	Troponin	MI diagnosis—acute MI	10,038 + 10,286 external—Secondary analysis of a stepped-wedge trial; external validation, 7 prospective cohorts	AUC 0.95; more ruled out at presentation
Toprak 2024 [20]	Point-of-care troponin	Rapid rule-out—acute MI	2560—Retrospective analysis of 2 prospective cohorts (US, Australia); external	Rule-out 35.1% vs. 15.2%/13.8% (ESC/ACC 0 h); NPV 99.96%
Guo 2023 (multiplex) [6]	Multiplex immunoarray (cTnI/H-FABP/copeptin)	Early MI—acute MI	260—Single-center proof of concept; internal, 10-fold CV	Outperformed troponin alone early (proof of concept)
Kusunose 2020 [10]	Echo	Wall-motion abnormality—regional dysfunction after prior MI	400 + 40—Retrospective; internal test set (n = 80) and independent-site validation (n = 40, AUC 0.90)	AUC 0.99; comparable to cardiologists, >trainees
Upton 2022 [21]	Stress echo	Severe CAD—anatomical obstructive CAD	reader study—Development on prospectively collected UK multicenter data (n = 578); randomized crossover reader study; external, retrospective US test set (n = 154)	Higher accuracy, agreement, confidence
Lin 2022 [22]	CCTA	Plaque + risk—anatomical plaque burden	9 cohorts—Retrospective, 9 cohorts at 11 sites; external validation cohort (n = 175)	IVUS-correlated; independent prognostic value
Nurmohamed 2024 [23]	CCTA	Vessel-specific ischemia—functional ischemia	CREDENCE → PACIFIC—Post hoc analysis of 2 prospective studies; external validation	Diagnoses FFR-ischemia; prognostic
Zhang 2019 [9]	CMR (non-contrast)	Chronic infarction—established MI (scar)	212 + 87—Retrospective single-center, case–control (212 MI vs. 87 controls); internal	AUC 0.94; sizing comparable to gadolinium
Betancur 2018 [24]/Otaki 2022 [25]	SPECT MPI	Obstructive CAD—anatomical obstructive CAD	1638/3578—Retrospective multicenter registry (REFINE SPECT; Otaki also 1519 conventional-camera patients); Betancur internal 10-fold CV, Otaki + external test (n = 555)	AUC 0.80/0.83 (Otaki internal; 0.80 external); >perfusion deficit; Otaki > readers
Zhang 2026 [26]	PET MPI	Obstructive CAD—anatomical obstructive CAD	1664 of 17,348—Retrospective, 4 sites; external validation at 3 sites	AUC 0.83; >experienced physicians
McKeen 2025 (ECG-FM) [27]	ECG foundation model	Multiple downstream—multiple; not ischemia-specific	1.5M ECGs—Retrospective; held-out institutional cohort + public benchmark	Label-efficient; >task-specific at low data
Li 2026 [28]	Magnetocardiography (edge AI)	Ischemia screening—functional ischemia	2118 (test 423)—Retrospective, single-center case–control; internal	Multi-view fusion of one magnetocardiographic signal; AUC 0.865, sensitivity 88.3%, specificity 66.5% (per cardiac cycle)

## Data Availability

No new data were created or analyzed in this study. Data sharing is not applicable to this article.

## Abbreviations

The following abbreviations are used in this manuscript:

ACC	American College of Cardiology
ACS	acute coronary syndrome
AI	artificial intelligence
AI-ECG	artificial intelligence-enhanced electrocardiogram
AUC	area under the receiver-operating-characteristic curve
CAD	coronary artery disease
CAD-RADS	Coronary Artery Disease Reporting and Data System
CCTA	coronary CT angiography
CI	confidence interval
CMR	cardiac magnetic resonance
CT	computed tomography
cTnI	cardiac troponin I
CV	cross-validation
ECG	electrocardiogram
ECG-FM	electrocardiogram foundation model
ESC	European Society of Cardiology
EU	Europe
FDA	US Food and Drug Administration
FFR	fractional flow reserve
H-FABP	heart-type fatty-acid-binding protein
IVUS	intravascular ultrasound
LLM	large language model
MI	myocardial infarction
MPI	myocardial perfusion imaging
NPV	negative predictive value
NSTEMI	non-ST-elevation myocardial infarction
OMI	occlusion myocardial infarction
PCI	percutaneous coronary intervention
PET	positron emission tomography
PROBAST	Prediction model Risk Of Bias Assessment Tool
QCA	quantitative coronary angiography
RCT	randomized controlled trial
RWMA	regional wall-motion abnormality
SPECT	single-photon emission computed tomography
STEMI	ST-elevation myocardial infarction
TIMI	Thrombolysis in Myocardial Infarction
UK	United Kingdom
US	United States
